# Synergism with Shikimic Acid Restores β-Lactam Antibiotic Activity against Methicillin-Resistant *Staphylococcus aureus*

**DOI:** 10.3390/molecules29071528

**Published:** 2024-03-29

**Authors:** Limin Hou, Minqi Ye, Xiaoyu Wang, Yifan Zhu, Xueyan Sun, Ruiheng Gu, Liangzhu Chen, Binghu Fang

**Affiliations:** 1 College of Veterinary Medicine, South China Agricultural University, Guangzhou 510642, China; 19120536126@163.com (L.H.); a172055990@163.com (M.Y.); 18238682167@163.com (X.W.); 15617098093@163.com (Y.Z.); 18737375927@163.com (X.S.); 19120536189@163.com (R.G.); 2 Guangdong Wenshi Dahuanong Biotechnology Co., Ltd., Yunfu 510610, China; 15514900126@163.com

**Keywords:** shikimic acid, methicillin-resistant *Staphylococcus aureus*, synergistic effect

## Abstract

Methicillin-resistant *Staphylococcus aureus* (MRSA) has evolved into a dangerous pathogen resistant to beta-lactam antibiotics (BLAs) and has become a worrisome superbug. In this study, a strategy in which shikimic acid (SA), which has anti-inflammatory and antibacterial activity, is combined with BLAs to restart BLA activity was proposed for MRSA treatment. The synergistic effects of oxacillin combined with SA against oxacillin resistance in vitro and in vivo were investigated. The excellent synergistic effect of the oxacillin and SA combination was confirmed by performing the checkerboard assay, time-killing assay, live/dead bacterial cell viability assay, and assessing protein leakage. SEM showed that the cells in the control group had a regular, smooth, and intact surface. In contrast, oxacillin and SA or the combination treatment group exhibited different degrees of surface collapse. q-PCR indicated that the combination treatment group significantly inhibited the expression of the *mec*A gene. In vivo, we showed that the combination treatment increased the survival rate and decreased the bacterial load in mice. These results suggest that the combination of oxacillin with SA is considered an effective treatment option for MRSA, and the combination of SA with oxacillin in the treatment of MRSA is a novel strategy.

## 1. Introduction

The emergence of antimicrobial resistance (AMR) can pose a threat to animal and world health, in particular *Staphylococcus aureus* (MRSA) [1]. MRSA is an opportunistic pathogen that is capable of colonizing humans and animals [2] and is known to cause multifarious infections in humans and animals [3]. Recently, MRSA strains were discovered in several animal-derived foods, such as poultry, pork, beef [4,5,6,7], and milk [8], indicating that animal-derived foods may be contaminated by MRSA [9]. MRSA causes a large number of diseases, including skin and soft tissue infections, bacteremia [10], endocarditis [11], pneumonia [12], and sepsis [13]. beta-lactam antibiotics (BLAs) are the most effective treatment for diseases and are widely used in animals [14]. Currently, MRSA is resistant to nearly all BLAs, limiting treatment options [15]. A major BLA determinant of MRSA is the expression of the *mec*A gene, which encodes penicillin binding protein 2A (PBP2a) [16]. MRSA generates insensitive affinity for PBP2a, which enables its related resistance to BLAs [11]. To date, combating resistance mechanisms against these BLAs is essential for restoring their antibacterial efficacy, which has proven highly impactful [17]. Combination drug therapy has become an effective strategy for controlling bacteria [18]. Therefore, identifying new agents or combinations of drugs for the treatment of MRSA infections is crucial [19].

Studies have indicated that some traditional Chinese medicines (TCMs) can reverse bacteria resistance [20]. Thus, TCM when combined with antibiotics plays a crucial role in bacterial infection treatment. TCMs combined with antibiotics restore the drug susceptibility of bacteria [21,22]. TCMs have often been chosen because of their high security and moderate price [23]. Increasing research results suggest that herbal extracts in combination with antibiotics can exhibit joint action against MRSA [24,25,26]. Previous research has shown the combined antimicrobial activity of *Polyalthia longifolia* leaf ethyl acetate fraction (PLEAF) with ampicillin against local MRSA [27]. Ampicillin in combination with ceftriaxone can reverse bacterial resistance against MRSA [28]. The in vitro potential activity of morin in combination with BLAs against MRSA indicates that the combination treatment of morin with BLAs is dependent on the PBP2a-mediated mechanism [29]. Therefore, exploring the combination of bioactive ingredients with antibiotics is an inevitable trend toward combating bacterial resistance [30].

Shikimic acid (SA) is a natural organic compound and an important intermediate in the biosynthesis of aromatic compounds in plants [31]. Studies have shown that SA has many biological activities, including antibacterial, anti-inflammatory, and antioxidant activities [32,33]. SA has been shown to be available in terms of action against *S. aureus* [34], and the effect of SA on the cellular functions of MRSA was investigated by measuring the intracellular pH, ATP concentration, and DNA content. The antibacterial activity and mechanism of SA in response to cell membrane damage have been investigated, and the results indicate that an obvious change in membrane action was observed [35]. Although the antibacterial activity of SA has been demonstrated, the concentration of SA on *S. aureus* is higher, and MRSA is more resistant to BLAs. Therefore, to solve these problems, we selected SA in combination with BLAs to reduce the dose of each drug, prevent drug resistance, and achieve greater antibacterial effects than the sum of the effects of single agents alone. However, the antibacterial mechanism of synergistic actions between BLAs and SA in combination with MRSA has not yet been reported, and the mechanism behind this synergistic effect is unclear. Therefore, the effect of the previously studied SA in combination with oxacillin was examined to investigate the antibacterial mechanism of a synergistic effect in treatment.

## 2. Result

### 2.1. Drug Sensitivity Measurement Results

**Figure 1:**
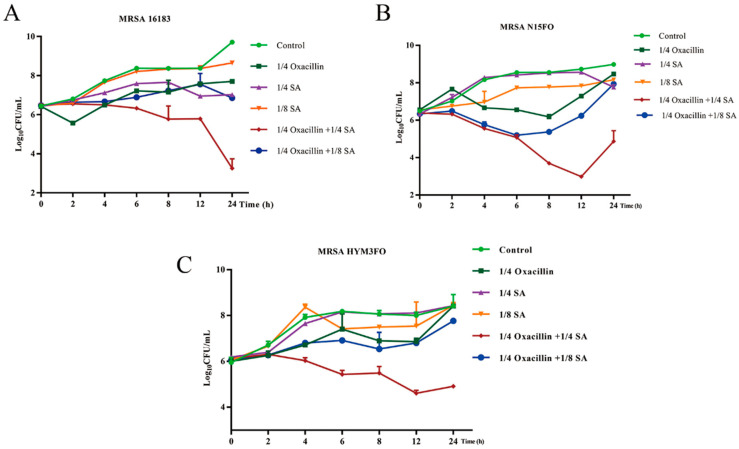
Time-killing kinetics of the synergistic combination of oxacillin with SA against (**A**) MRSA 16,183, (**B**) MRSA N15FO, and (**C**) MRSA HYM3FO.

As shown in Table 1, many *S*. *aureus* strains demonstrate resistance to BLAs. The MICs of the identified BLAs ranged from 1 to ≥1024 μg/mL. The MIC of SA was 4000 μg/mL for all the strains. The synergistic effects of the combinations of BLAs with SA on MRSA were tested using checkerboard assays. As shown, the FICIs of oxacillin combined with SA were lower than those of the other BLAs, and the synergy indices of oxacillin with SA ranged from 0.3125 to 0.5 for different clinical strains of MRSA. The FICIs of amoxicillin with SA ranged from 0.2578 to 1. The FICIs of ampicillin with SA ranged from 0.5 to 0.75. The FICIs of ceftriaxone, ceftiofur, cefoxitin, cefovecin, and ceftazidime with SA were 0.375, 0.5, 0.5, 0.75, and 0.625, respectively. In this study, we ultimately chose oxacillin in combination with SA for the treatment of MRSA via in vitro and in vivo antimicrobial tests based on the FICI. To further investigate the synergistic effect of oxacillin and SA against MRSA, time-kill assays were used. MRSA 16,183, N15FO, and HYM3FO were selected for the time-kill assay. The concentrations of oxacillin and SA were selected from the checkerboard method with FICIs ≤ 0.5. The oxacillin concentration was 1/4 × MIC, and the SA concentrations were 1/4 and 1/8 × MIC. As shown in Figure 1A–C, the control group and the 1/8 × MIC SA treatment groups of MRSA 16,183 exhibited almost no rapid growth inhibition, after which the effects were less significant or negligible. Notably, 1/4 × MIC oxacillin in combination with 1/4 × MIC SA significantly decreased growth (4-log_10_ CFU/mL) after 12–24 h compared with 1/4 × MIC oxacillin alone for MRSA 16,183, N15FO, and HYM3FO.

### 2.2. Effects on Cell Ultrastructure

**Figure 2:**
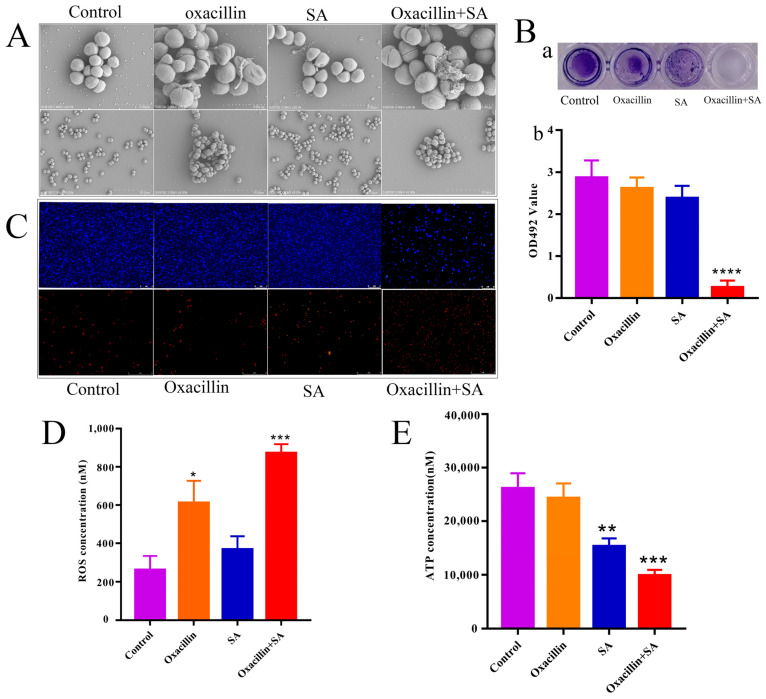
The effect of oxacillin and SA on bacterial structure and apoptosis through exhibited antibiofilm activity, as well as measurements of ROS and ATP levels. (**A**) Bacterial morphology affected by oxacillin, SA, or a combination group compared to the control group (2 μm and 10 μm). (**B**) Biofilms was treated by 1% crystal violet for combination oxacillin with SA. Biofilm formation was quantified by measuring sample absorbance at a wavelength of 595 nm and percentages were calculated with the untreated biofilm as the basis. (**C**) Bacterial apoptosis affected by oxacillin, SA, or a combination group compared to the control group (0.01016 mm). (**D**) Measured ROS. (**E**) Measured ATP. Values are presented as mean ± SD. * *p* < 0.05 was considered to indicate significant difference, ** *p* < 0.01, *** *p* < 0.001 and **** *p* < 0.0001 were considered to indicate an extremely significant difference.

SEM was used to observe the ultrastructural changes in bacteria upon exposure to the oxacillin and SA combination. As shown in Figure 2A, cells in the control group had a regular, smooth, and intact surface. In contrast, oxacillin and SA and the combination treatment group exhibited different degrees of surface collapse. In the control group, the cell wall and membrane were intact, and the wall was defined. However, SEM analysis of the cells exposed to oxacillin alone or in combination showed that the cell membrane and wall became fuzzy. The effects of oxacillin in combination with SA on MRSA film formation were determined via crystal violet staining. As shown in Figure 2B, compared with oxacillin or SA alone, oxacillin and SA in the combination group significantly inhibited biofilm formation (*p* < 0.0001). To further investigate whether the cell membrane and wall were damaged, live/dead staining of MRSA was performed. Live/dead straining of MRSA was applied to determine the bactericidal capacity of oxacillin in combination with SA. DAPI stains all cells and emits green fluorescence, while PI emits red fluorescence dye and selectively stains dead cells. As shown in Figure 2C, in the control group or in the presence of oxacillin or SA alone, almost no red fluorescence was observed, indicating that most of the bacteria were still alive. Compared with the control group, a small amount of red fluorescence was detected in the oxacillin and SA treatment group, indicating that these substances have an effect on antibacterial activity. However, for oxacillin in combination with SA, the amount of red fluorescence was significantly greater than in the groups with oxacillin or SA alone, indicating that the percentage of dead cells was greater than that in the groups with oxacillin or SA alone. Therefore, the combination oxacillin with SA has an obvious bactericidal effect by fluorescence microscope. And then, the effect of oxacillin in combination with SA on ROS and ATP generation in MRSA was also examined. As shown in Figure 2D, after exposure to oxacillin in combination with SA for 1 h, a large amount of ROS was produced in *S. aureus*. Moreover, the ROS level in the oxacillin with SA combination group was significantly greater than that in the single-agent group. For oxacillin–SA, the ROS levels were significantly elevated, which confirm med that oxacillin–SA plays an important role in antibacterial activity. ATP is a direct cellular energy source, and the level of ATP reflects cell energy metabolism and survival. As shown in Figure 2E, the ATP level was significantly lower in the combination group (*p* < 0.001) than in the oxacillin and SA groups. The results indicate that the combination of oxacillin and SA has a synergistic bactericide effect on MRSA.

### 2.3. Effects of the MRSA Membrane and Expression of the mecA Gene

**Figure 3:**
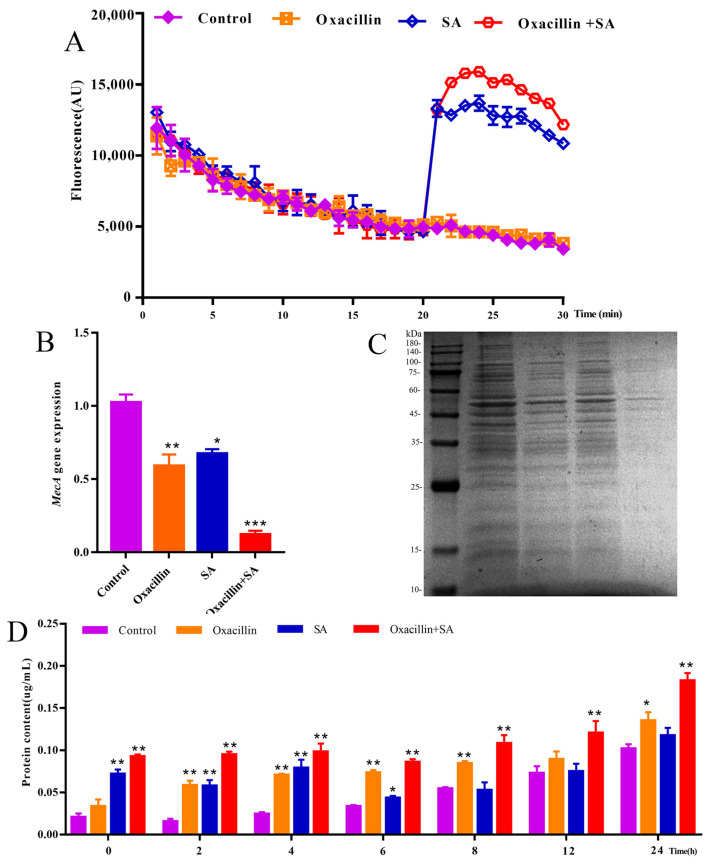
Assessment of the combination of oxacillin with SA in terms of exerting antibiofilm effects and regulating the expression of *Mec*A genes, as well as results of the membrane permeability assay. (**A**) Analysis of oxacillin and SA or their combination in terms of the depolarization effects of the bacterial plasma membrane on MRSA. (**B**) q-PCR analysis of expression of the *mec*A gene. (**C**) The bacteria cells of protein were separated using sodium dodecyl sulfate-polyacrylamide gel electrophoresis (SDS-PAGE) and for the oxacillin–SA combination treatment group. (**D**) Verification of protein leakage using a BCA protein assay kit. Values are presented as mean ± SD. * *p* < 0.05 was considered to indicate significant difference, ** *p* < 0.01, ***p < 0.001 was considered to indicate an extremely significant difference.

To explore the potential mechanism underlying the combination of oxacillin and SA, we evaluated the cell membrane permeability of MRSA. As shown in Figure 3A, there were significant changes in fluorescence in the combination and SA treatment groups at 20 min. Compared to the control and oxacillin alone treatment groups, fluorescence intensity in the combination group was greater than that in the SA alone group. Therefore, the results indicate that the combination treatment had a certain impact on membrane permeability. As shown in Figure 3B, compared with those in the control group, the expression levels of *mec*A genes in the combination treatment group were downregulated approximately five-fold. In the oxacillin and SA treatment groups, the *mec*A gene was downregulated approximately two-fold. These results indicate that combination treatment significantly diminished bacterial virulence, and SA could be used in combination with BLAs to restore the efficacy of these agents against MRSA infection. To further investigate these results, the leakage of proteins was detected. The effects of protein leakage were analyzed by SDS-PAGE and a BCA protein assay kit. As shown in Figure 3C, the SDS-PAGE results revealed slight protein bands in the combination group compared to those in the control, oxacillin alone, and SA alone groups. The result suggested that the amount of protein leakage significantly increased with oxacillin with SA in combination treatment for MRSA. Moreover, as shown in Figure 3D, the BCA protein assay kit was used to detect protein leakage in the combination group, which was visually greater than that in the single-agent group at 0–24 h. Hence, protein leakage was analyzed via SDS-PAGE, and a BCA protein assay kit was used to determine that the intracellular protein concentration in the combination treatment group decreased, indicating that oxacillin in combination with SA can damage the cell membrane and leakage of protein macromolecules.

### 2.4. Oxacillin and SA Protect Mice from S. aureus Bacteremia

**Figure 4:**
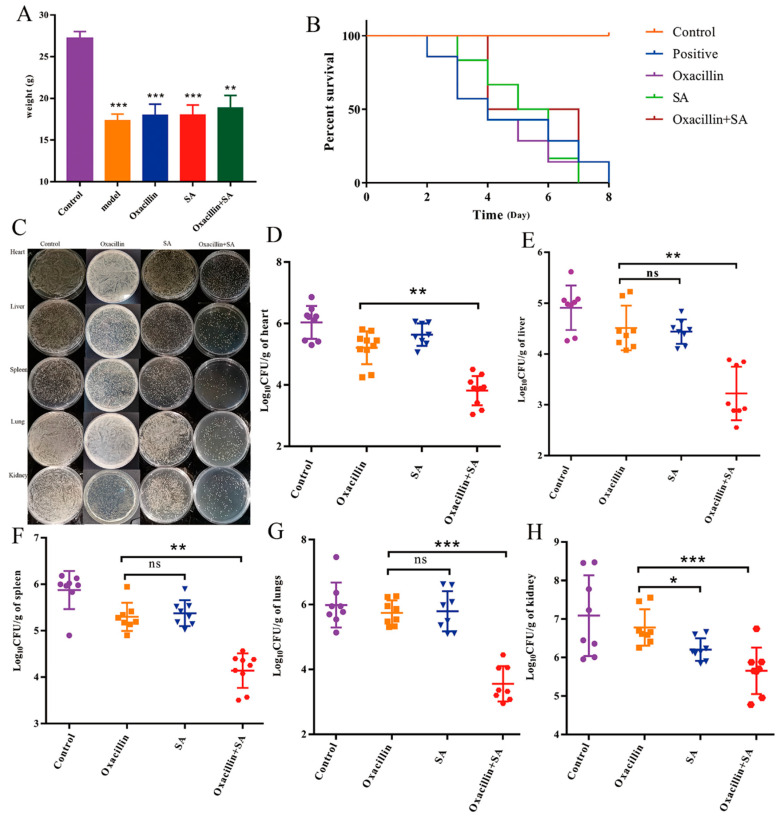
Oxacillin and SA in combination exerted promising therapeutic potential in this bacteremia model. (**A**) The effects of different treatment groups on the body weight of mice. (**B**) The survival rate of mice in the different treatment groups. (**C**) Plate count was used to observe the bacterial burden of the heart, liver, spleen, lungs, and kidneys in the control, model, oxacillin alone, SA alone, and combination treatment groups. (**D**) The bacterial burden of the hearts for oxacillin alone, SA alone, and their combination. (**E**) The bacterial burden of the livers for oxacillin alone, SA alone, and their combination. (**F**) The bacterial burden of the spleens for oxacillin alone, SA alone, and their combination. (**G**) The bacterial burden of the lungs for oxacillin alone, SA alone, and their combination. (**H**) The bacterial burden of the kidneys for oxacillin alone, SA alone, and their combination. The “ns” was mean no obvious change, * *p* < 0.05 was considered to indicated a statistically significant difference, ** *p* < 0.01, *** *p* <0.001 were considered to indicate an extremely significant difference.

**Figure 5:**
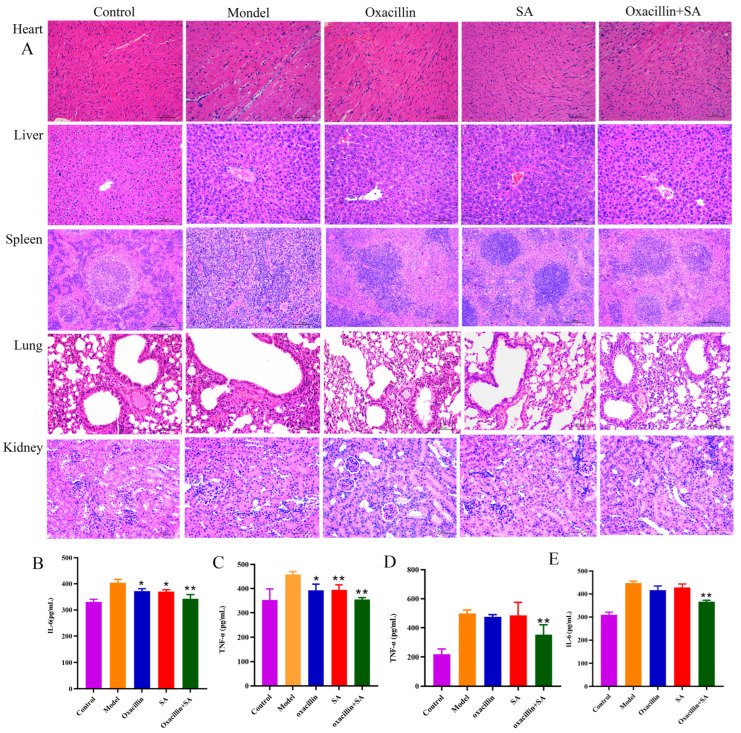
(**A**) HE staining was used to observe pathological changes in the heart (20 μm), liver (20 μm), spleen (50 μm), lungs (20 μm), and kidneys (5 μm) in the control, model, oxacillin alone, SA alone, and combination treatment groups. (**B**) The expression of the inflammatory factor IL-6 in the lungs. (**C**) The expression of the inflammatory factor TNF-α in the lungs. (**D**) The expression of the inflammatory factor TNF-α in the kidneys. (**E**) The expression of the inflammatory factor IL-6 in the kidneys. * *p* < 0.05 was considered to indicated a statistically significant difference, and ** *p* < 0.01 was considered to indicate an extremely significant difference.

Based on these in vitro findings, we further studied the protective effects of oxacillin and SA in vivo on mice with *S. aureus*-related bacteremia. Το investigate the therapeutic activity of oxacillin and SA in the bacteremia model, we performed and measured the body weight (Figure 4A) and survival assay (Figure 4B). As shown in Figure 4A, the weights of the control, model, oxacillin, SA, and combination groups were 27.20 ± 0.82, 17.69 ± 1.12, 18.3 ± 0.56, 18.02 ± 1.28, and 18.87 ± 1.47, respectively. Eight days after infection with 1 × 10^8^ CFUs of bacteria, the survival rate of mice in the infection group was 41%, that in the oxacillin and SA alone therapeutic group survival rates was 50%, and that in the survival rate of combination group was 83%. As shown in Figure 4B, the survival rate of mice in the combination group significantly increased by 30%, compared to that in the oxacillin and SA treatment groups after 8 days, which indicates that the SA combination treatment improved the survival rate of mice infected with acute bacteremia and that this treatment exhibited synergistic effects on the survival rate of mice infected with oxacillin. Moreover, after bacteremia was established, the body weights of the five groups of mice were monitored for 3 days. The bacterial burdens in the heart, liver, spleen, lung, and kidney were quantified to evaluate the influence of oxacillin on MRSA survival within the heart, liver, spleen, lung, and kidney. As shown in Figure 4C–H, oxacillin and SA significantly reduced the number of viable MRSA 16,183 cells in the heart, liver, spleen, lungs, and kidneys of the mice. For the untreated infected mice, the colony counts of the heart, liver, spleen, lung, and kidney were 6.03 ± 0.53 log_10_ CFU/g, 4.90 ± 0.43 log_10_ CFU/g, 5.87 ± 0.40 log_10_ CFU/g, 5.86 ± 0.90 log_10_ CFU/g, and 7.08 ± 1.04 log_10_ CFU/g, respectively. For the oxacillin treatment group, the colony counts of the heart, liver, spleen, lung, and kidney were 5.96 ± 0.50 log_10_ CFU/g, 4.51 ± 0.43 log_10_ CFU/g, 5.29 ± 0.30 log_10_ CFU/g, 5.74 ± 0.38 log_10_ CFU/g, and 6.85 ± 0.45 log_10_ CFU/g, respectively. For the SA treatment group, the colony counts of the heart, liver, spleen, lung, and kidney were 5.35 ± 0.42 log_10_ CFU/g, 4.42 ± 0.23 log_10_ CFU/g, 5.37 ± 0.27 log_10_ CFU/g, 5.79 ± 0.61 log_10_ CFU/g, and 6.16 ± 0.29 log_10_ CFU/g, respectively. For the combination treatment group, the colony counts of the heart, liver, spleen, lung, and kidney were 4.38 ± 0.46 log_10_ CFU/g, 3.15 ± 0.53 log_10_ CFU/g, 4.14 ± 0.37 log_10_ CFU/g, 3.66 ± 0.59 log_10_ CFU/g, and 5.66 ± 0.56 log_10_ CFU/g, respectively. The number of heart, liver, spleen, lung, and kidney bacteria significantly decreased to 1.58 ± 0.76 log_10_ CFU/g, 1.35 ± 0.76 log_10_ CFU/g, 1.16 ± 0.73 log_10_ CFU/g, 2.08 ± 1.07 log_10_ CFU/g, and 1.19 ± 0.65 log_10_ CFU/g, respectively. To assess the effect of oxacillin and SA treatment on the pathological manifestations of heart, liver, spleen, lung, and kidney injury, a histopathological study was carried out. Subsequently, the bacterial counts in the heart, liver, spleen, lung, and kidney tissue and further pathological changes associated with bacteremia were also assessed. The visual observation and pathological alterations of the HE staining are shown in Figure 5A. There were no pathological changes in the control group, and the model group had moderate to severe inflammation. In the hearts of the model group, as well as the oxacillin and SA treatment groups, cell nuclei were clearly vacuolized; there was severe pyknosis and myocardial fibrosis to various degrees, and the combination treatment significantly alleviated the cell nuclei through vacuolization and pyknosis. The liver tissue (from the model, oxacillin alone, and SA alone groups) exhibited different degrees of liver cell necrosis, excessive inflammation, disruption of the hepatic cell cords, and inflammatory cell infiltration. The combination treatment group exhibited hepatocyte vacuolation and a decreased number of inflammatory cells. The spleen tissue (model, oxacillin alone, and SA alone groups) showed markedly irregular architecture and massive neutrophil infiltration in the splenic corpuscles, and the combined group exhibited a small amount of neutrophil infiltration. Compared to the combination group, the lung tissues in the model, oxacillin alone, and SA alone groups showed severe morphological lesions, such as alveolar septa that were widened with obvious hyperemia and dilation of capillaries. Hematoxylin and eosin (HE) staining of kidney sections (model, oxacillin alone, and SA alone treatment groups) demonstrated glomerular atrophy, necrosis, and increased inflammatory cell infiltration, and the combination treatment resulted in slight glomerular atrophy. The process of bacterial infection is often accompanied by bursts of inflammatory responses [36]. As shown in Figure 5B–E, the combination of oxacillin with SA significantly inhibited the MRSA-induced production of IL-6 and TNF-α.

## 3. Discussion

Antibiotic resistance has long been a worldwide health problem causing major threats due to the overuse or misuse of antibiotics [37]. The widespread use of BLAs has led to the emergence of multidrug resistant bacteria, especially MRSA. Therefore, developing a new effective pathway to decrease the resistance of MRSA to BLAs is necessary, and novel antimicrobial agents for combating MRSA are urgently needed. Compared to antibiotics, SAs have attracted greater attention due to their easy availability, low cost, pharmacological activity, and minimal side effects [38]. To date, no one has reported their use in combination with antibiotics against MRSA. To our knowledge, this study is the first to demonstrate that SA combined with oxacillin has synergistic effects both in vivo and in vitro, providing a new therapeutic option for clinical bacteremia. To fully understand the antimicrobial mechanism of the combination of BLAs with SA against MRSA, we explored SA in combination with BLAs in the treatment of MRSA as a novel strategy.

Checkerboard and time-kill curve analyses are common methods used to evaluate the synergistic effects of different drugs [39]. After combination with SA, the MICs of the BLAs for three of the strains were reduced by one to eight times compared with those of the BLAs alone. Notably, the lowest FICI was observed for the oxacillin and SA combination group. Our studies revealed that a lower synergistic concentration of the combination could inhibit MRSA in vitro. With increasing concentrations of SA, the results of the kill-time assay were approximately >3 log_10_ CFU/mL lower than those of the single-agent treatment at 12–24 h for MRSA 16,183, N15FO, and HYM3FO. Thus, the combination of these synergistic effects with the MRSA strains tested could suggest that the results are concentration dependent. These data indicate that SA has a low degree of resistance development, highlighting its potential in combating MRSA infection.

The formation of biofilm protects bacteria from host immune responses, antibiotics, and chemotherapies, making infection intervention extremely difficult [40]. A previous study indicated that SA is related to membrane structure and affects bacterial cell division [31]. With respect to SA, which is an organic acid, studies have shown that organic acids can enter the bacterial cell membrane and can damage enzymes and cell structures [41]. In this study, the OD values of the control, oxacillin alone, SA alone, and combination treatment groups were 3.06 ± 0.01, 2.93 ± 0.14, 3.03 ± 0.01, and 0.11 ± 0.01, respectively. The results show that the combination of oxacillin with SA significantly inhibited the formation of bacterial biofilm, which was more strongly inhibited by combination treatment than by the control treatment. To further investigate whether cell death was related to the membrane structure, live/dead cells were detected [42]. The combination treatment of oxacillin with SA caused a large number of MRSA deaths. Consistent with the findings of previous studies, SA was shown to damage the membrane of cells [43]. Therefore, oxacillin in combination with SA markedly altered membrane integrity, leading to intracellular protein leakage and the promotion of cell death. These results suggest that inhibiting the formation of biofilms on MRSA may be one of the mechanisms underlying the synergistic effect of these bacteria.

In normal cells, the production of ROS approximately balances antioxidant defense mechanisms [44]. However, when ROS production exceeds antioxidant defenses, oxidative stress occurs [45]. ROS function as signaling molecules in various pathways regulating both cell survival and cell death [46]. The combination of the two agents in the treatment of MRSA caused cell apoptosis by inducing the formation of excessive ROS. ATP is an enzyme in the cytoplasm that leaks into the periplasmic space [47]. In the present study, compared with the other three groups (control, oxacillin, and SA), the ATP concentration of MRSA in the extracellular–intracellular environment in the combination therapy group decreased after combination therapy. These results suggest that the combination of oxacillin with SA caused damage to MRSA. The decrease in ATP could be mainly due to a decrease in ATP synthesis and an increase in ATP hydrolysis [48]. In the present study, the combination of oxacillin with SA markedly disrupted membrane integrity, upregulated the level of ROS, and downregulated the level of ATP and the *mec*A gene. The ROS generated may subsequently cause oxidative damage, DNA damage, and cellular damage. To summarize, the antibacterial mechanism of the inhibitory action of oxacillin in combination with SA on MRSA may involve effects on membrane integrity, the leakage of protein, and DNA and cellular damage.

Bacteremia is a bacterial infection that occurs in the bloodstream, and S. aureus is one of the most common pathogens that generates many different infections [49]. It has been reported that SA has anti-inflammatory and antibacterial effects, and compared with previous studies, this agent was nontoxic to the mice at the applied dosage in the present study. The bacterial load of tissue is an important indicator for assessing antimicrobial effects in vivo. In this study, oxacillin and SA worked synergistically, and their effects on bacterial load and survival rate were observed. We observed that the combination treatment had the most effective antibacterial effect on MRSA–bacteremia infection. The results of this study suggest that bacteremia cannot be treated directly with oxacillin due to the resistance of MRSA. The treatment effect in the combination group was greater than that in the single treatment group, as were pathological changes and the fungal burden in the tissue. IL-6 and TNF-α are cytokines that are promptly and transiently produced in response to infections and tissue injuries. They play an important role in host defense and cell death by stimulating acute phase responses. It is crucial to detect IL-6 and TNF-α expression in disease infection. Moreover, IL-6 and TNF-α enable augmentation of inflammatory cells in areas of local infection, increasing the effects of other cytokines and inflammation. In the present study, the concentrations of IL-6 and TNF-α in the lung and kidney tissues of different groups of mice were detected. The results suggested that the combination treatment significantly decreased the levels of IL-6 and TNF-α in the lung and kidney and further indicated that the bacterial load was lower in the combination treatment group than in the single treatment group. The protective effect may be related to the anti-inflammatory effects of SA. 

## 4. Materials and Methods

### 4.1. Materials and Chemicals

Shikimic acid and all antibiotics, including oxacillin, amoxicillin, ampicillin, ceftiofur, carbenicillin, and cefoxitin, were purchased from Macklin (Shanghai, China). PI, DAPI, the ATP Assay Kit, the ROS Assay Kit, and the Alkaline Phosphatase Assay Kit, were purchased from Beyotime (Shanghai, China). The ELISA Kit was purchased from Elkbiotechnology (Denver, CO, USA).

### 4.2. Bacterial Strains

The methicillin-resistant *Staphylococcus aureus* (MRSA) strains used *S*. *aureus* 16,183, N15FO, and HYM3FO obtained from Associate Professor Yufeng Zhou of the School of Veterinary Medicine, South China Agricultural University (Guangzhou, China).

### 4.3. Minimal Inhibitory Concentration (MIC)

The MICs were determined in triplicate using the microdilution method in a 96-well plate, as previously described [50]. Each experiment was independently repeated three times.

### 4.4. Synergy Assay

A checkerboard assay was performed to determine the synergistic effects of SA with BLA-related antibiotics against *S. aureus*, and inhibitory effects were visualized to calculate the fractional inhibitory concentration index (FICI); FICIs ≤ 0.5 were considered synergistic [51].

### 4.5. Time-Killing Growth Curve Assay

The curves of the bacterial-killing effect on the three types of MRSA were investigated via a modified method, as previously described [52]. The time-killing growth of bacterial curves was plotted with time as the X axis and CFU/mL as the Y axis.

### 4.6. Scanning Electron Microscopy (SEM)

SEM was used to observe bacterial cell morphology. Colonies from the MRSA 16,183 culture grown on MH agar (16–18 h) were transferred to MH broth. The SEM analysis was carried out as described previously [53].

### 4.7. Live/Dead Bacterial Cell Viability Assay

PI and DAPI staining were used for the live/dead bacterial cell viability assay [54]. Fluorescence microscopy (Bio-Rad, Hercules, CA, USA) was then used to observe and record the live/dead fluorescence levels.

### 4.8. Biofilm Formation Inhibition Assay

Analysis of biofilm formation in *S. aureus* strains was performed using crystal violet analysis. For the biofilm inhibition assay, the protocol was used as reported previously [55].

### 4.9. Reactive Oxygen Species (ROS) Measurement

DCFH-DA reagent was used to detect intracellular ROS according to a previous method [56]. A microplate reader was used to measure the OD value.

### 4.10. Measurement of Extracellular–Intracellular ATP

Intracellular ATP levels were measured according to a previous method [57].

### 4.11. mRNA Expression of Penicillin-Binding Protein (PBP)

Total RNA was extracted from MRSA using an RNA extraction kit (Vazyme, Nanjing, China). Relative gene expression levels were measured using the 2^ΔΔCt^ method [58].

### 4.12. Measurement of Protein Leakage

The integrity of the cytomembrane was determined by SDS-PAGE and a BCA protein kit [59]. A spectrophotometer was used to measure the absorbance at 595 nm to determine protein leakage.

### 4.13. Membrane Permeability Assay

As previously described with minor modifications, the cell membrane potential-sensitive fluorescent dye propidium iodide was used to detect the effect of combinations of oxacillin and SA on *S*. *aureus* 16,183 cell membrane permeability [60]. For details on the operating steps, please refer to the instructions concerning PI.

### 4.14. Murine Model of Bacteremia

All animal experimental procedures were reviewed and approved by the South China Agricultural University Institutional Animal Ethics Committee. Female KM mice aged 8 weeks were used to establish a bacteremia model and were then arbitrarily divided into five groups (*n* = 8 per group): the model group, oxacillin group, SA group, antibiotic combination groups, and the control group. MRSA 16,183 (1 × 10^8^ CFUs) was administered as the bacteria. After administration for 72 h, the mice were sacrificed via eye vein blood collection. Bacterial loads in the liver, spleen, heart, lung, kidney, and stool were quantified. Histopathology and changes in inflammatory factors were observed.

## 5. Conclusions

In summary, the combination of oxacillin with SA had excellent antibacterial effects. Our study revealed that the antibacterial effect of the combination treatment was more potent than individual treatments in vitro and in vivo. These results indicate that SA or oxacillin may not be suitable for direct use as anti-MRSA drugs in vitro and in vivo and should instead be considered ancillary drugs for oxacillin. These findings suggest the usefulness of treatment methods in the clinic and indicate that further research may lead to significant breakthroughs. We herein demonstrated the combination effect against MRSA in vitro and in vivo. These findings are important for the development of therapeutics and provide a basis for new treatment options for treating MRSA.

## Figures and Tables

**Table 1:** FICI values for β-lactam antibiotics against *S. aureus*.

Antibiotic	Strain	MIC_antibiotic_ (μg/mL)	FIC	MIC_SA_ (μg/mL)	FIC	FICI	Interpretation
Amoxicillin	N21	512	0.25	4000	0.25	0.5	Synergistic
	43,300	32	0.0078	8000	0.25	0.2578	Synergistic
	N30	512	0.25	8000	0.5	0.75	Additive
	N24	512	0.25	8000	0.25	0.5	Synergistic
	N22	512	0.5	4000	0.5	1	Irrelevant
Oxacillin	16,183	128	0.0625	4000	0.25	0.3125	Synergistic
	N27FO	4	0.25	4000	0.25	0.5	Synergistic
	N15FO	2	0.25	4000	0.25	0.5	Synergistic
	HYM3FO	4	0.25	4000	0.25	0.5	Synergistic
Ampicillin	S16-5′	16	0.5	4000	0.25	0.75	Additive
	43,300	8	0.125	4000	0.25	0.375	Synergistic
	S14-19	128	0.25	4000	0.25	0.5	Synergistic
Ceftriaxone	S14-19	4	0.25	4000	0.125	0.375	Synergistic
Ceftiofur	16,183	128	0.5	4000	0.25	0.5	Synergistic
Cefoxitin	16,183	64	0.25	4000	0.25	0.5	Synergistic
Cefovecin	16,183	1024	0.5	4000	0.25	0.75	Additive
Ceftazidime	16,183	128	0.5	4000	0.125	0.625	Additive

## Data Availability

Data are contained within the article.
